# Supplementary material for: A Novel Rodent Model of Hypertensive Cerebral Small Vessel Disease with White Matter Hyperintensities and Peripheral Oxidative Stress
Source: Int J Mol Sci. 2022 May 25;23(11):5915. doi: 10.3390/ijms23115915 (PMC9180536; doi:10.3390/ijms23115915)
Supplement: Supplementary file 1 [file ijms-23-05915-s001.zip › ijms-1720560-supplementary.pdf]

International Journal of
Molecular Sciences

Supplementary Materials:

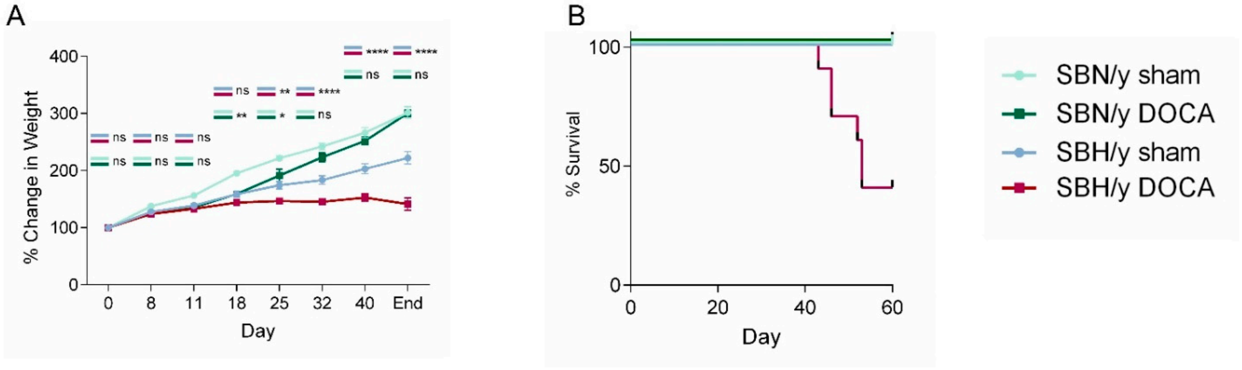

Figure S1: Growth curve. Growth rate normalized to baseline body weight of SBH/y sham (*n=8*), SBH/y-DOCA (*n=11*), SBN/y sham (*n=4*) and SBN/y-DOCA (*n=4*) rats **(A)**. *$*P < 0.05$, $**P < 0.01$, $***P < 0.001$, $****P < 0.0001$*. Two-way ANOVA.

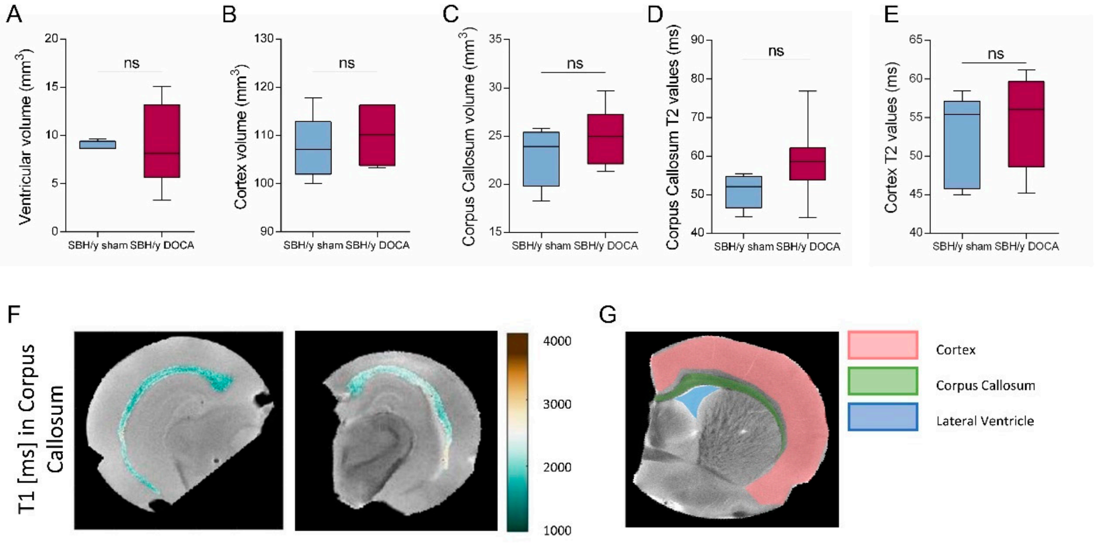

Figure S2: MRI measures and masks. Volume of ventricle **(A)**, cortex **(B)** and corpus callosum **(C)**, and average T2 values of cortex **(D)** and corpus callosum **(E)** of SBH/y sham (*n=6*) and SBH/y-DOCA (*n=9*) rats. Representative T1 values of corpus callosum of SBH/y sham and SBH/y DOCA brains **(F)**. Example of masks used to analyze MRI images **(G)**. Boxplot presents the median and interquartile range, the whiskers show extreme data points, two-tailed t-test in **(A-E)**.

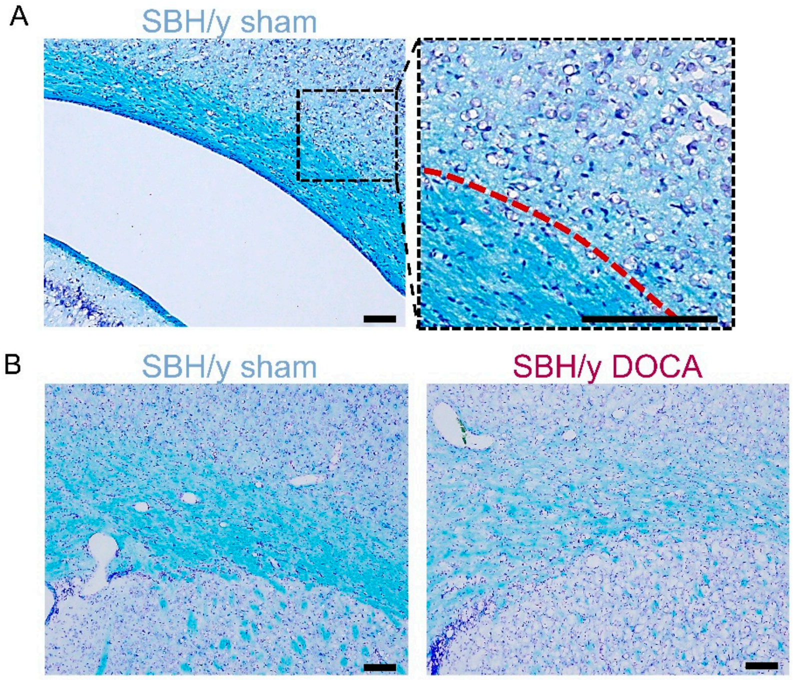

Figure S3: White matter staining of SBH/y sham and SBH/y-DOCA brains. Representative luxol blue and cresyl violet staining of SBH/y sham and 5x magnification of the white matter-grey matter interface **(A)**. Spotted red line identify the border between the white matter and the grey matter. Another representative luxol blue and cresyl violet staining of SBH/y sham and SBH/y-DOCA brains, indicating on white matter pathology **(B)**. Scale bars = 200 µm.
